# Effects of a Community Intervention on HIV Prevention Behaviors among Men Who Experienced Childhood Sexual or Physical Abuse in Four African Settings: Findings from NIMH Project Accept (HPTN 043)

**DOI:** 10.1371/journal.pone.0099643

**Published:** 2014-06-13

**Authors:** Joseph Daniels, Arnošt Komárek, Tawanda Makusha, Alastair Van Heerden, Glenda Gray, Alfred Chingono, Jessie K. K. Mbwambo, Thomas Coates, Linda Richter

**Affiliations:** 1 University of California Los Angeles, UCLA Center for World Health, Los Angeles, California, United States of America; 2 Faculty of Mathematics and Physics, Charles University, Prague, Czech Republic; 3 Human Sciences Research Council, Durban, South Africa; 4 University of the Witwatersrand/Chris Hani Baragwanath Hospital, Faculty of Health Sciences, Perinatal HIV Research Unit, Soweto, South Africa; 5 University of Zimbabwe, College of Health Sciences, Harare, Zimbabwe; 6 Muhimbili University of Health and Allied Sciences, Muhimbili University Teaching Hospital, Dar es Salaam, Tanzania; 7 Developmental Pathways to Health Research Unit, University of the Witwatersrand, Johannesburg, South Africa; Indiana University and Moi University, United States of America

## Abstract

**Background:**

There is increased focus on HIV prevention with African men who report experiencing childhood sexual (CSA) or physical abuse (CPA).

**Objective:**

To better understand the effects of a community-based intervention (Project Accept HPTN 043) on HIV prevention behaviors among men who report CSA or CPA experiences.

**Methods:**

Project Accept compared a community-based voluntary mobile counseling and testing (CBVCT) intervention with standard VCT. The intervention employed individual HIV risk reduction planning with motivational interviewing in 34 African communities (16 communities at 2 sites in South Africa, 10 in Tanzania, and 8 in Zimbabwe). Communities were randomized unblinded in matched pairs to CBVCT or SVCT, delivered over 36 months. The post-intervention assessment was conducted using a single, cross-sectional random survey of 18-32 year-old community members (total N = 43,292). We analyzed the effect of the intervention on men with reported CSA or CPA across the African sites. Men were identified with a survey question asking about having experienced CSA or CPA across the lifespan. The effect of intervention on considered outcomes of the preventive behavior was statistically evaluated using the logistic regression models.

**Results:**

Across the sites, the rates of CSA or CPA among men indicated that African men reflected the global prevalence (20%) with a range of 13–24%. The statistically significant effect of the intervention among these men was seen in their increased effort to receive their HIV test results (OR 2.71; CI: (1.08, 6.82); P: 0.034). The intervention effect on the other designated HIV prevention behaviors was less pronounced.

**Conclusion:**

The effect of the intervention on these men showed increased motivation to receive their HIV test results. However, more research is needed to understand the effects of community-based interventions on this group, and such interventions need to integrate other keys predictors of HIV including trauma, coping strategies, and intimate partner violence.

## Background

There is increased focus on understanding HIV prevention among African men who report having experienced sexual or physical abuse as children [1], [2]. Most research on the effects of childhood sexual (CSA) and physical abuse (CPA) on HIV risk has focused on the most vulnerable populations such as poor women, commercial sex workers, injecting drug users and those with mental illness; but there has been limited research with men, bar men who have sex with men. Predictors of HIV risk behaviors among those with a history of CSA or CPA include intersecting issues like trauma, shame, limited coping strategies and substance abuse [3]–[5]. A history of CSA has indicated increased HIV risk behavior during adulthood including intimate partner violence [1], [6]–[10]. Intimate partner violence increases the risk of HIV, and most men who perpetrate such violence have experienced physical violence as a child and rely on particular masculine social norms that privilege and protect men's actions [1], [2], [11]–[13]. Clinical studies have shown that psycho-educational, coping and stress reduction interventions are effective in reducing HIV risk behaviors within this group [4]–[12]. Yet more research is needed to understand community-based interventions in reducing HIV risk behaviors among men with a history of childhood sexual or physical abuse living in African settings.

### Objective

We sought to measure the effectiveness of Project Accept, a community-based voluntary counseling and testing (CBVCT) intervention, on HIV risk behavior change among men who have experienced childhood sexual or physical abuse. We do not focus on men who have perpetuated such abuses here. This report describes the results of an analysis using data from control and intervention communities collected in three African countries that included four geographically separated sites. This paper elaborates on the effectiveness of an intervention that included a risk-reduction voluntary counseling and testing model coupled with motivational interviewing for HIV prevention behavior change. Previous analysis of Project Accept data has shown that CBVCT is effective in recruiting youth, men and women in rural areas as well as those who experienced childhood sexual or physical abuse [1], [14]. Utilizing a multi-assay algorithm method, the HIV prevalence was determined to be 16.5% with a 1.60% incidence rate among participants in this study [15]. Overall, the study showed a 13.9% reduction in HIV incidence in CBVCT communities compared to standard VCT communities along with increased testing among CBVCT [16].

## Methods

This study was conducted in partnership with community advisory boards and local government departments. The study was the National Institutes of Mental Health Project Accept, HPTN 043 [16], [17]. The study was granted ethical approval by all U.S. and African institutional review boards including: Charles University, Chiang Mai University Research Institute for Health Sciences, Fred Hutchison Cancer Research Center, Human Sciences Research Council (South Africa), International Center for Research on Women, John Hopkins Bloomberg School of Public Health, John Hopkins University School of Medicine, Medical University of South Carolina, Muhimbili University of Health and Allied Sciences, National Institute of Allergy and Infectious Diseases, University of California Los Angeles and San Francisco, University of North Carolina Chapel Hill, University of Southampton (Southampton), University of Witwatersrand (Chris Hani Baragwanath Hospital), University of Zimbabwe. Verbal informed consent was obtained for the post-intervention behavioral assessment prior to participation in that component of the study. With the permission of the IRBs overseeing the study at all sites, verbal informed consent for these study components was accepted, rather than written informed consent, as a signed consent form would link the participant to the study and could jeopardize participant confidentiality. The consent procedures were approved by all of the ethics committees/IRBs.

Project Accept was a cluster-randomized trial in three African countries (South Africa, Tanzania, Zimbabwe) and Thailand, implemented from 2005-2011. Baseline demographic, behavioural, and social data were collected from a random sample of community residents 18 and 32 years of age [18]. This was followed by community randomization and a 36-month intervention period [19]. The impact of the study intervention was assessed across entire communities in a single cross-sectional survey conducted at the end of the intervention period. Demographic and behavioural data and blood samples were collected from randomly-selected individuals.

The CBVCT intervention was designed to change community and individual responses to HIV by removing barriers such as fees, inconvenience, waiting time for results, fear and stigmatization. The intervention utilized four components: community mobilization, easy access to VCT, post-test support services, and real-time performance feedback to reach study targets. The community mobilization utilized outreach coordinators and workers to encourage discussion of HIV, disclosure of HIV status, increase uptake of testing and reduce stigma. Mobile HIV testing units provided easy access to VCT services. Support groups, as post-test services, were provided to anyone who had tested regardless of HIV status. These groups included topic discussions on HIV risk, treatment, testing, disclosure and services. The real time performance feedback tracked progress toward achieving pre-set study goals.

The intervention communities in each site were paired with equal numbers of control communities. The control communities received standard HIV voluntary counseling and testing services at primary health care clinics. The study was implemented in Pwani, an agricultural area of Tanzania with a high Muslim population; Mutoko, a rural area of Zimbabwe; and two sites in South Africa, Soweto and Vulindlela. Soweto is a diverse, urban area adjacent to Johannesburg, and Vulindlela is mainly Zulu-speaking community that is a mixed urban and semi-rural area of KwaZulu-Natal.

Baseline and post-intervention behavioral surveys were conducted within each community. Researchers administered the survey, and participants were allowed to skip responses to questions based on previous answers to questions in the survey. The baseline assessment was conducted before randomization and did not include HIV testing (because it was part of the intervention). The post-intervention assessment was independent of the baseline assessment. Households were selected with equal probability from all enumerated households in the community. One participant was randomly requested from each selected household to complete the baseline assessment. At the post-intervention assessment, all individuals from selected households were invited to contribute blood samples, and one person was randomly selected for a detailed demographic and behavioral questionnaire.

The analysis presented in this paper makes use of post-intervention data only. The baseline and post-intervention surveys included 130-items for demographics, alcohol and drug use, sexual risk behavior, history of HIV testing and disclosure, social norms and HIV and AIDS stigma. CSA and CPA were assessed with two questions: “When you were growing up (before 12 years old), did you undergo any unwanted sexual/physical abuse?” Outcome items included last HIV test, received results of HIV test, partner disclosure, condom use, and talked about HIV within the last six months. The surveys were developed in English, translated into local languages, and then piloted in each community before being finalized for administration.

The effect of intervention on considered outcomes of the preventive behavior was statistically evaluated using logistic regression models and R software [20]. The intervention effect was quantified by the odds ratios where OR>1 means that the intervention (community based VCT) increases probability of the preventive behavior compared to the standard VCT. The following analyzes were performed: (i) by site; (ii) to test whether the intervention has a homogeneous effect across sites; (iii) an overall (“All sites”) analysis, e assuming that the intervention effect is the same across the sites. All odds ratios are adjusted for the effect of paired communities. The overall all sites odds ratios are further adjusted for the effect of sites.

## Results

### Sample Characteristics

The estimated total size of the population in each site for this study was 76,300 in Zimbabwe, 54,900 in Tanzania, 66,100 in Vulindlela, and 152,500 in Soweto. The median household size ranged from three in Soweto to five in Zimbabwe, with four in Tanzania and Vulindlela. Response rates for the post-intervention survey were 93.6% in Tanzania, 84.7% in Vulindlela, 84.5% in Soweto, and 84% in Zimbabwe. Denominators in the results section vary by: a) the number of people who were eligible to answer the question as pre-determined by questionnaire skip patterns, and b) the number of eligible individuals who answered the question.

The age, education, work, and marriage status characteristics of the samples for this analysis from each of the four settings are shown in Table 1. Both men with and without reported CSA or CPA are represented, and we note that both groups of men are comparable suggesting that there is a limited influence of background characteristics on the intervention.

**Table 1:** Demographic characteristics among men at post-intervention assessment.

Total	Soweto, SA	Vulindlela, SA	Zimbabwe	Tanzania
(N = 4788)	(N = 1349)	(N = 1125)	(N = 1131)	(N = 1183)
**Men with CSA or CPA, n (%)**				
	287 (21.4)	192 (17.2)	260 (23.0)	165 (13.9)
	N = 1344	N = 1114	N = 1130	N = 1183
**Among men with CPA or CSA**				
**Education in Years, n (%)**				
<5	2 (0.7)	7 (3.7)	12 (4.6)	27 (16.9)
5–7	6 (2.1)	11 (5.9)	54 (20.8)	93 (58.1)
8–10	54 (18.8)	54 (28.7)	72 (27.7)	22 (13.8)
11–12	148 (51.6)	114 (60.6)	104 (40.0)	16 (10.0)
>12	77 (26.8)	2 (1.1)	18 (6.9)	2 (1.2)
	N = 287	N = 188	N = 260	N = 160
**Age, n (%)**				
18–22	112 (39.0)	104 (55.0)	111 (42.7)	76 (46.1)
23–27	98 (34.1)	59 (31.2)	74 (28.5)	38 (23.0)
28–32	77 (26.8)	26 (13.8)	75 (28.8)	51 (30.9)
	N = 287	N = 189	N = 260	N = 165
**Married, n (%)**				
	16 (5.6)	0 (0.0)	93 (35.8)	46 (27.9)
	N = 285	N = 187	N = 260	N = 165
**Employed, n (%)**				
	190 (66.2)	98 (51.9)	186 (71.5)	135 (81.8)
	N = 287	N = 189	N = 260	N = 165
**Among men without CPA or CSA**				
**Education in Years, n (%)**				
<5	4 (0.4)	12 (1.3)	19 (2.2)	117 (12.5)
5–7	18 (1.7)	46 (5.1)	148 (17.1)	576 (61.6)
8–10	173 (16.4)	232 (25.5)	204 (23.5)	117 (12.5)
11–12	611 (58.0)	586 (64.5)	436 (50.3)	112 (12.0)
>12	248 (23.5)	33 (3.6)	60 (6.9)	13 (1.4)
	N = 1054	N = 909	N = 867	N = 935
**Age, n (%)**				
18–22	470 (44.5)	471 (51.5)	383 (44.0)	376 (37.0)
23–27	322 (30.5)	282 (30.9)	271 (31.1)	245 (24.1)
28–32	263 (24.9)	161 (17.6)	216 (24.8)	396 (38.9)
	N = 1055	N = 914	N = 870	N = 1017
**Married, n (%)**				
	46 (4.4)	8 (0.9)	306 (35.2)	389 (38.3)
	N = 1051	N = 911	N = 869	N = 1016
**Employed, n (%)**				
	656 (62.3)	389 (42.7)	549 (63.2)	816 (80.2)
	N = 1053	N = 910	N = 868	N = 1017

Overall, men reporting either CSA or CPA represented 18.8% (N = 4788) of all men participating in Project Accept. Men who reported either CSA or CPA ranged from 21.4% in Soweto, 17.2% in Vulindlela, 23.0% in Zimbabwe and 13.9% in Tanzania. Most men who reported abuse had 11–12 years of education with 51.6% in Soweto, 60.6% in Vulindlela, and 40.0% in Zimbabwe, but most men in Tanzania had 5–7 years (58.1%) of education. There was a low representation of tertiary education reported across the sites. Higher numbers of men reported being married in Zimbabwe and Tanzania at 35.8% and 27.9% respectively, compared to marriage numbers of 5.6% in Soweto and 0% in Vulindlela. These differences in marriage rates between South African and non-South African sites may be explained by men's low financial capacity to pay bride price to get married [21]. However, falling marriage rates have been observed since at least the 1950s, a trend that has continued through recent decades [22], [23]. The employment rate among these men was 66.2% Soweto, 51.99% Vulindlela, 71.5% Zimbabwe, and 81.8% Tanzania. The demographic rates of men reporting either CSA or CPA were similar to the demographic rates among men not reporting these experiences when compared within sites.

In Table 2, the rates of HIV preventative behavior among the control and intervention groups are presented for each of the sites. Five items from the survey are used to define HIV preventive behavior. These items ask participants if they: 1) talked about HIV in the last 6 months; 2) were tested for HIV in the last 12 months; 3) received their HIV test results; 4) told their partner(s) about the results; and 5) always wore a condom. There was a generally equal representation of men who experienced CSA or CPA in both the intervention and control groups per setting. The range of rates for CSA or CPA in the intervention and control groups across all the settings was 13.5–24% and 14.4–21.9% respectively. Zimbabwe had the highest reported rates in each group.

**Table 2:** Rates of post-intervention preventive behavior among CSA or CPA men.

Total	Soweto, SA		Vulindlela, SA		Zimbabwe		Tanzania	
(N = 4788)	(N = 1349)		(N = 1125)		(N = 1131)		(N = 1183)	
	Control	Intervention	Control	Intervention	Control	Intervention	Control	Intervention
**Men with CSA or CPA, n (%)**								
	146 (21.4)	141 (21.3)	101 (18.9)	91 (15.7)	139 (24.0)	121 (21.9)	81 (13.5)	84 (14.4)
	N = 682	N = 662	N = 535	N = 579	N = 578	N = 552	N = 601	N = 582

**Among men with CPA or CSA**								
**Talked about HIV in past 6 months, n (%)**								
	107 (79.3)	97 (77.6)	67 (82.7)	51 (72.9)	63 (64.3)	57 (69.5)	35 (66.0)	34 (56.7)
	N = 135	N = 125	N = 81	N = 70	N = 98	N = 82	N = 53	N = 60
**Tested for HIV in past 12 months, n (%)**								
	42 (65.6)	38 (57.6)	28 (68.3)	28 (58.3)	22 (66.7)	32 (74.4)	18 (64.3)	23 (56.1)
	N = 64	N = 66	N = 41	N = 48	N = 33	N = 43	N = 28	N = 41
**Received the result of the last HIV test, n (%)**								
	50 (90.9)	52 (96.3)	28 (80.0)	41 (97.6)	28 (93.3)	38 (95.0)	16 (80.0)	31 (88.6)
	N = 55	N = 54	N = 35	N = 42	N = 30	N = 40	N = 20	N = 35
**Told partner the result of the HIV test, n (%)**								
	39 (83.0)	41 (87.2)	21 (84.0)	24 (77.4)	15 (60.0)	26 (81.2)	14 (87.5)	17 (70.8)
	N = 47	N = 47	N = 25	N = 31	N = 25	N = 32	N = 16	N = 24
**Always used condom, n (%)**								
	28 (58.3)	28 (59.6)	9 (39.1)	24 (66.7)	12 (57.1)	12 (37.5)	7 (46.7)	13 (48.1)
	N = 48	N = 47	N = 23	N = 36	N = 21	N = 32	N = 15	N = 27

The most significant rates were noted within the item, ‘receiving HIV test results’ and show low ‘usage of condoms all the time’. Rates of men in the intervention group telling their partner about their HIV test results was above 70% except for the control group in Zimbabwe. Although the rates for receiving HIV test results were relatively high across the sites and groups, the rates of HIV testing within the last 12 months was lower, but above 56% across the control and intervention groups among the sites. The same is true for talking about HIV in the last six months. Always using condoms was higher in the intervention groups across the sites except for Zimbabwe where 37.5% of the men reported always using condoms within the intervention group compared to the reported 57.1% within the control group. Additionally, a logistic regression was run to evaluate the effect of the intervention within each site and across the sites within men who reported experiencing CSA or CPA.

### HIV prevention behavior change within control and intervention groups

In Table 3, we show the odds ratios depicting the effect of the Project Accept intervention on the HIV preventive behavior among men who reported childhood sexual or physical abuse: odds ratio, 95% confidence interval, P-value.

**Table 3:** Effect of intervention on preventive behavior among CSA or CPA men.

	Soweto, SA (N = 287)	Vulindlela, SA (N = 192)	Zimbabwe (N = 260)	Tanzania (N = 165)	Homogeneity of OR's across sites	All sites (N = 904)
**Talked about HIV in past 6 months**						
	0.88 (0.48, 1.61)	0.51 (0.22, 1.14)	1.25 (0.66, 2.37)	0.71 (0.32, 1.58)	P: 0.366	0.84 (0.60, 1.19)
	P: 0.667	P: 0.100	P: 0.496	P: 0.396		P: 0.336
**Tested for HIV in past 12 months**						
	0.79 (0.38, 1.65)	0.69 (0.27, 1.77)	1.81 (0.62, 5.25)	0.74 (0.27, 2.07)	P: 0.532	0.88 (0.56, 1.39)
	P: 0.523	P: 0.437	P: 0.278	P: 0.568		P: 0.580
**Received the result of the last HIV test**						
	3.24 (0.56, 18.67)	5.25 (0.54, 51.38)	1.32 (0.16, 11.02)	2.34 (0.45, 12.00)	P: 0.842	**2.71 (1.08, 6.82)**
	P: 0.189	P: 0.155	P: 0.797	P: 0.310		P: **0.034**
**Told partner the result of the HIV test**						
	1.29 (0.39, 4.22)	0.59 (0.12, 2.91)	2.48 (0.72, 8.52)	0.41 (0.07, 2.50)	P: 0.327	1.14 (0.58, 2.24)
	P: 0.673	P: 0.513	P: 0.150	P: 0.335		P: 0.712
**Always used condom**						
	1.14 (0.49, 2.66)	**4.41 (1.15, 16.90)**	0.43 (0.13, 1.40)	1.10 (0.27, 4.52)	P: 0.090	1.17 (0.68, 2.04)
	P: 0.758	P: **0.030**	P: 0.163	P: 0.892		P: 0.568

Odds ratio, 95% confidence interval, P-value. All odds ratios are adjusted for effect of paired communities. The overall *All sites* odds ratios are further adjusted for the effect of sites.

In separate analyzes by site, the only significant effect of intervention is seen for the item ‘always used condom’ in Vulindlela where intervention significantly increases probability of using condoms. For all items except ‘always used condom’, the test of homogeneity of odds ratios across sites is highly non-significant, suggesting that it is possible to assume the same intervention effect across sites. For this reason, the results of the combined sites were analyzed. This reveals a significant effect of intervention on ‘received the result of the last HIV test’ item where intervention significantly increases the probability of receiving the result of the last HIV test. The test of homogeneity of the odds ratios across sites for item ‘always used condoms’ also is not significant at a 5% level. However, it is already significant at a 10% significance level. Hence we suggest that the interpretation of the all sites odds ratio for ‘always used condoms’ with a care since we do not have strong evidence for the same intervention effect across sites.

## Discussion

In this paper we attempt to further understand how a community-based voluntary counseling and testing program influenced HIV preventive behaviors among men who experienced childhood sexual or physical abuse. These preventive behaviors were: talk about HIV within the last 6 months; test for HIV within the last 12 months; received the test result; told partner the result; and used condoms all the time. The study demonstrated that a high proportion of men across the African sites in Project Accept reported experiencing childhood sexual and physical abuse. These rates of CSA and CPA men found here complement findings of a 20% prevalence of CSA and CPA among men and women globally [5]. In Project Accept, this post-intervention analysis provides needed insight on the representation of CSA and CPA among men in a cluster-randomized trial and the effect of a community-based intervention on HIV risk behavior within this group [1], [2].

Although efficacy studies have measured improved HIV prevention behaviors among those who experienced CSA or CPA, this is one of the first analyses conducted on a community-based survey to understand similar effects [10], [12]. The major finding from this analysis was that the intervention conducted within the Project Accept was effective in increasing the proportion of men who received their HIV test results as part of individual health planning. We conclude that this community-based approach to voluntary counseling and testing did influence men knowing their status by getting their HIV test results. However, there was not a strong measured effect of the intervention on the other HIV prevention behaviors assessed. As a result, more research is needed to examine the effects of such interventions at the community level. In particular, these interventions need to consider how intersecting predictive variables such as trauma, coping and interpersonal violence in adulthood contribute to HIV risk and how these can be explicitly integrated into individual risk reduction plans [10], [12]. Ultimately, CSA and CPA among African men should be considered in HIV prevention strategies that include counseling and testing.
